# Some Cases of Disease of the Heart, with an Inquiry into Their Nature and Causes

**Published:** 1818-06

**Authors:** 


*Some Cases of Disease of the Heart, with an Inquiry into their Nature and Causes.* By J. H. James, Esq. Surgeon to the Exeter Hospital.

(Medico-Chirurgical Transactions, Vol. viii.)

Both the pathology and physiology of the heart and vascular system are yet in their infancy. It is only by an accumulation of facts that the *former* can be improved. The cases here recorded, were patients in Bartholomew's Hospital, where the author had opportunities of seeing the symptoms when living, and the appearances after death.

Case 1. *(Case 4 in the Volume, as the three first are mere dissections a few days after entering the hospital.)*

A man, 34 years of age, was admitted into hospital early in the spring of 1812, apparently in a dying state. Countenance and lips livid and swollen; eyes red and suffused; breathing excessively difficult; cough with hæmoptysis; great debility and emaciation; slight œdema of the feet; pulse scarcely perceptible at the wrist, and extremely irregular; strong pulsation of the heart and carotids; ability to lie low, and on either side; bowels irregular; appetite lost; urine high coloured and scanty. Lead was given to check the hæmoptysis; squill to increase the urine. For some time it was doubtful whether he would live or die; but his bowels becoming open, his state improved, especially when the urine increased in quantity. He was discharged six weeks after his entry, reprieved rather than cured.

In the month of August he returned in nearly the same state as when he first entered; his legs much swollen. He died in a short time. On dissection it was found, that the heart had undergone no other alteration "than the simple augmentation of the size of its cavities."

The cases as well as the reasonings are rather inconclusive; and consequently we shall pass them over very superficially.

Case 2. *(Case 7 in Volume.)* 29th of February, 1812, a young woman presented herself, without the appearance of having suffered from previous illness; the account she gave of herself being this, that three days before, she had fallen down stairs, and struck the lower part of the sternum against the edge of one of them. She had got up again, without assistance, but had been ill from that time. She was now sitting up in bed, breathing laboriously, with countenance flushed and suffused; pulse accelerated, but not much disturbed; tongue slightly furred; nervous system little affected; had spat some blood. Venesection afforded no relief; the pulse became smaller, more frequent, and irregular; died March 3d. *Dissection.* The cavity of the thorax as full as possible of serum; lungs compressed into a narrow space. The heart was actively dilated in size; the foramen ovale open, as if it had recently burst.

Case 3. Ann Hogan, ætat 48, admitted 26th of March for ascites. Had been tapped the preceding autumn for the same complaint. Was again tapped on the 9th of May. *Symptoms at the time of report.* Abdomen very tumid, with evident fluctuation; digestive functions much impaired. The thoracic functions not much disturbed. She could fairly lie back in bed; pulse frequent, but not remarkable for any thing else. She died four days after being tapped. *Dissection* shewed scarcely any cavity belonging to the left ventricle, while its parietes were remarkably thick and strong. The annulus venosus was much contracted.

The scope of Mr. James's observations tends to make it appear that enlargements of the heart are very frequently, if not generally, owing to obstruction of the circulation in the minute, that is, the nutrient and secerning vessels of the body. That obstruction in any portion of the circuit which the blood makes between leaving and returning to the heart must derange the function of the circulating organ, can admit of little doubt. The plethora which results from deficient secretion too, must cause the central organ considerable embarrasment. But, after all, we believe that structural diseases of the heart are owing to the same general causes which produce morbid changes in other internal organs; and that metastases of irritation from the surface and from other viscera are frequent causes of its organic diseases.

